# Reconstituting Microtubules: A Decades-Long Effort From Building Block Identification to the Generation of Recombinant α/β-Tubulin

**DOI:** 10.3389/fcell.2022.861648

**Published:** 2022-04-28

**Authors:** Shih-Chieh Ti

**Affiliations:** School of Biomedical Sciences, Faculty of Medicine, The University of Hong Kong, Pokfulam, Hong Kong SAR, China

**Keywords:** tubulin, microtubules, tubulin isotypes, tubulin protein biochemistry, recombinant tubulin

## Abstract

Microtubules are cytoskeletal filaments underlying the morphology and functions of all eukaryotic cells. In higher eukaryotes, the basic building blocks of these non-covalent polymers, ɑ- and β-tubulins, are encoded by expanded tubulin family genes (i.e., isotypes) at distinct loci in the genome. While ɑ/β-tubulin heterodimers have been isolated and examined for more than 50 years, how tubulin isotypes contribute to the microtubule organization and functions that support diverse cellular architectures remains a fundamental question. To address this knowledge gap, *in vitro* reconstitution of microtubules with purified ɑ/β-tubulin proteins has been employed for biochemical and biophysical characterization. These *in vitro* assays have provided mechanistic insights into the regulation of microtubule dynamics, stability, and interactions with other associated proteins. Here we survey the evolving strategies of generating purified ɑ/β-tubulin heterodimers and highlight the advances in tubulin protein biochemistry that shed light on the roles of tubulin isotypes in determining microtubule structures and properties.

## Introduction

α/β-tubulin heterodimers polymerize into microtubules that are fundamental to various cellular processes, including cell division, migration, and organelle transport [reviewed in (Nogales, 2000)]. However, not all cells form microtubules with the same composition. Cells can express multiple tubulin isotypes that are different from each other in amino acid sequences (Ludueña and Banerjee, 2008). Humans have at least nine α- and ten β-tubulin isotypes (Findeisen et al., 2014), and most of them can acquire a variety of post-translational modifications, including acetylation, polyglutamylation, and de-tyrosination (Janke and Bulinski, 2011). This diversity in tubulin is important; specialized cells, such as neurons, often express specific tubulin isotypes (Ludueña and Banerjee, 2008), and accumulation of α-tubulin acetylation is a marker for long-lived, stable microtubules (Janke and Bulinski, 2011). Exactly how heterogeneous microtubule composition is established and used by cells to facilitate functional outputs is still an open question.

**FIGURE 1:**
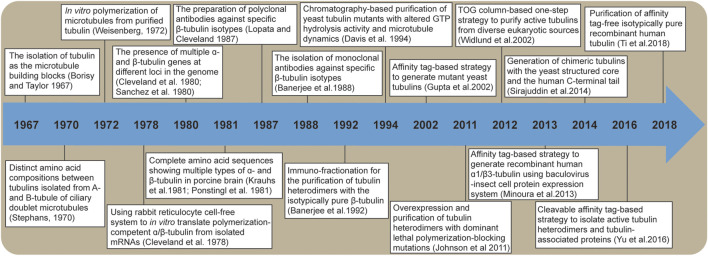
A timeline of major breakthroughs that have advanced our understanding of tubulin isotypes.

*In vivo* genetics and cell biology studies have revealed the critical roles of tubulin isotypes and tubulin post-translational modifications in forming functional cellular microtubule architectures (i.e., the tubulin code) (Sullivan, 1988; Wilson and Borisy, 1997; Verhey and Gaertig, 2007; Janke and Bulinski, 2011). In particular, among tubulin variants that cause phenotypes in a wide variety of eukaryotes, mutations in specific tubulin isotypes have been associated with human diseases such as neurological disorders, impaired oocyte maturation, and defective platelet formation (Gadadhar et al., 2017; Pham and Morrissette, 2019). However, the challenge of generating biochemically pure tubulin has limited our ability to reconstitute microtubules with a defined tubulin composition for quantitative *in vitro* biochemical and biophysical characterization. How tubulin isotypes determine microtubule properties (e.g., dynamics and post-translational modifications) remains unclear. This review focuses on the milestones in protein biochemistry that have advanced our understanding of microtubule biology (Figure 1). From the initial isolation of α/β-tubulin protein heterodimers as the building block of microtubules, the characterization of purified tubulin variants, to the recent breakthrough in generating recombinant tubulin, the decades-long effort is now ready to decipher the molecular mechanisms underlying the biological functions of tubulin isotypes.

### The Isolation of “Tubulin” as the Building Blocks of Microtubules

**FIGURE 2:**
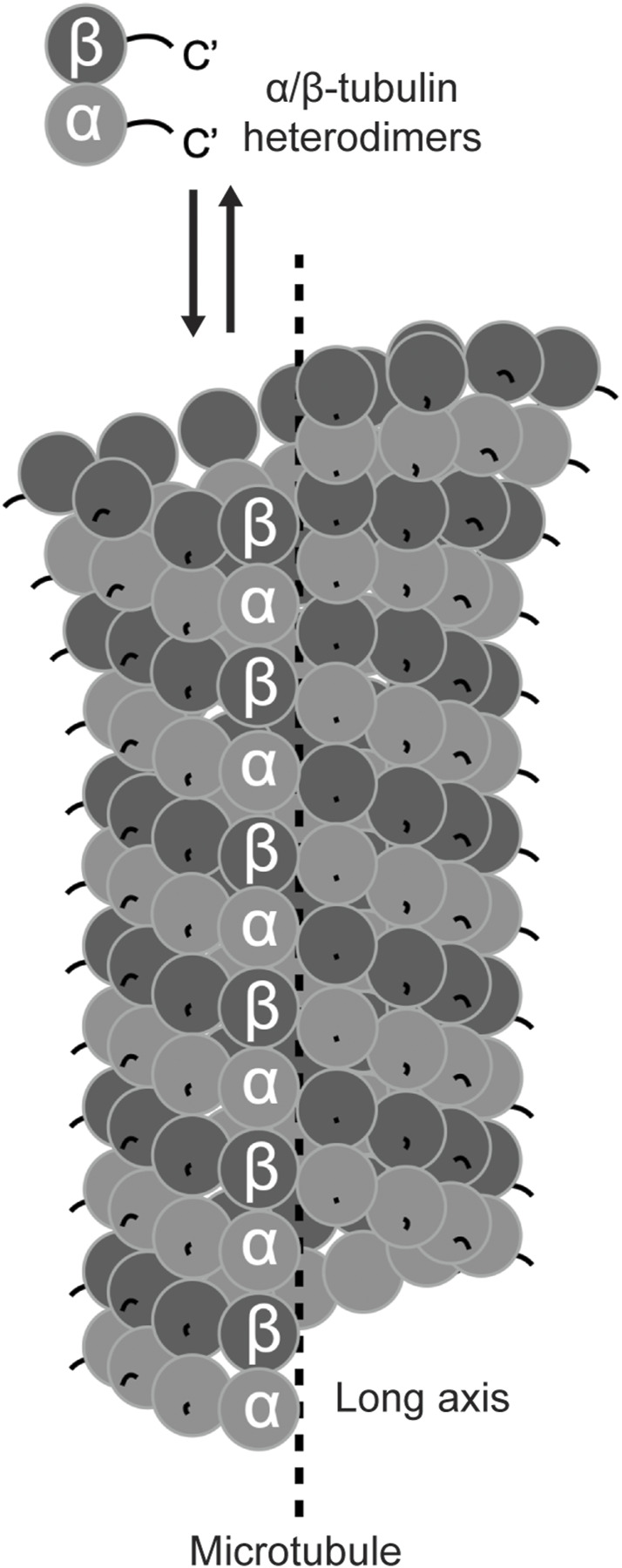
Schematic of α/β-tubulin heterodimers and a microtubule. The structured cores of α- (light grey) and β- (dark grey) tubulin are shown in circles. The unstructured C-terminal tails are exposed on the surface of the microtubule.

In the early 1960s, negative stain electron microscopy images observed the ubiquitous tubular filaments (i.e., microtubules) in diverse cell types across species (Ledbetter and Porter, 1963; Slautterback, 1963). Further examinations, with improved fixing reagents and negative staining strategies, described microtubules as thirteen beaded profibrils surrounding the long axis of the filaments (Pease, 1963; Ledbetter and Porter, 1964; Gall, 1966; Phillips, 1966) (Figure 2). While these fine features are coherent to the modern structural model of microtubules, identifying the building blocks was challenging, mainly due to the lack of effective assays for tracing a microtubule-associated property during biochemical fractionation of the cell lysate.

Colchicine disrupts diverse cellular functions without inhibiting DNA, RNA, and protein synthesis (Taylor, 1965). To reveal the mechanism of action, Borisy and Taylor tracked the radioactivity of tritium-labeled colchicine in fractionated cell homogenates. They found that colchicine targeted a 6S protein enriched in cells or tissues with abundant microtubules, suggesting the colchicine-binding protein to be a subunit of the filaments (Borisy and Taylor, 1967). The following early characterization of the microtubule subunit proteins depended on studies using cilia and flagella, which have the unique 9 + 2 arrangement of microtubules that can be solubilized and fractionated in mild conditions (Gibbons and Grimstone, 1960; Gibbons, 1963). Guided by colchicine-binding activity as well as the electron micrographs of cilia and flagella, the microtubule building blocks were isolated as a dimeric protein of two 55 kDa components, and each protein dimer bound two molecules of guanine nucleotide and one molecule of colchicine (Shelanski and Taylor, 1967, 1968; Stephens et al., 1967; Renaud et al., 1968). Further analyses identified the microtubule subunits as heterodimers of two different kinds of proteins, α- and β-tubulin (Bryan and Wilson, 1971; Feit et al., 1971; Olmsted et al., 1971). With the name ‘tubulin’ (Mohri, 1968), the purified microtubule subunit proteins have led to *in vitro* biochemical and biophysical studies providing mechanistic insights into diverse cellular processes, such as cell division, cell migration, and intracellular cargo transport.

### The Formulation of the “Multi-Tubulin Hypothesis”

Compared to the colchicine-binding protein isolated from the mammalian brain, the purified cilia and flagella tubulin showed the similarity of microtubule subunits in molecular weight, amino acid composition, and guanine nucleotide-binding activity (Weisenberg et al., 1968). This study not only revealed the conservation of microtubule building blocks but also established the critical knowledge (e.g., time sensitivity, the necessity of Mg-GTP, and limited exposure to high salt) for employing brain tissues as the source to purify a large quantity of active tubulin. In particular, the rapid exchange of one tubulin-bound guanine nucleotide with free nucleotides confers the necessity of including GTP during the purification process to maintain the native conformation of tubulin (Weisenberg et al., 1968).

The initial characterization indicated the biochemical similarity in tubulin isolated from different sources. However, the variations in the stability of cellular architectures when subjected to chemical (e.g., colchicine) and physical (e.g., exposure to non-physiological temperature) treatment suggested microtubules with different tubulin compositions. The evidence supporting this hypothesis originated from ciliary and flagellar cold-resistant doublet microtubules, where A-tubules had higher thermostability than B-tubules and remained intact when treated with the elevated temperature (Behnke and Forer, 1967). Temperature-dependent fractionation (i.e., thermal fractionation) of flagellar doublet microtubules revealed the distinct amino acid compositions between tubulins isolated from A- and B-tubules (Stephens, 1970). Further study demonstrated the presence of different types of tubulin in cilia and neuronal cells (Olmsted et al., 1971). These early biochemical studies formulated the ‘multi-tubulin hypothesis’ that the diversity of tubulin proteins underlies the formation of microtubule networks for various biological functions in cells (Fulton and Simpson, 1976).

### The Discovery of Tubulin Variants With Distinct Amino Acid Sequences

For several years after the success in tubulin isolation, it had been challenging to reconstitute and characterize the mechanisms of microtubule assembly and disassembly, which play a fundamental role in the cellular functions of the cytoskeletal filaments. To address this limitation, a critical study with detailed biochemical characterization recognized the key factors [magnesium ions (Mg^2+^), EGTA, and warm temperature (35°C)] stimulating *in vitro* microtubule assembly from purified tubulin proteins (Weisenberg, 1972). In contrast, elements like calcium ions (Ca^2+^), EDTA, and cold temperature (0°C) inhibited the formation of the filaments (Weisenberg, 1972). Further characterization identified a high concentration of glycerol [4 M, or about 37% (w/v)] as a reagent that conferred rapid nucleation and superior stability of the tubulin polymers, allowing the development of a strategy to purify tubulin proteins by reversible microtubule polymerization (Shelanski et al., 1973). With phosphocellulose chromatography removing the tubulin-associated proteins, the temperature-dependent microtubule polymerization and depolymerization provided a robust methodology for isolating a large quantity of brain tubulin (Borisy et al., 1975; Weingarten et al., 1975). Since then, purified mammalian brain tubulin (usually from porcine or bovine) has served as a popular material for *in vitro* assays to dissect the molecular basis of microtubule structure, polymerization dynamics, and interaction with microtubule-associated proteins. By improving the efficiency of *in vitro* microtubule polymerization, a recent protocol can generate tubulin with controlled post-translational modifications from cell lines or brain tissues of genetically engineered mice (Souphron et al., 2019).

Cycles of microtubule assembly and disassembly have been the core of protocols for obtaining tubulin from non-neuronal cell lines (Doenges et al., 1977; Nagle et al., 1977; Weber et al., 1977; Weatherbee et al., 1978; Doenges et al., 1979; Newton et al., 2002) as well as from different tissues of a variety of species, such as sea urchin (Binder and Rosenbaum, 1978; Farrell and Wilson, 1978; Keller et al., 1982; Detrich and Wilson, 1983), fungi (Kilmartin, 1981; Yoon and Oakley, 1995; Braun et al., 2009; Drummond et al., 2011), nematodes (Dawson et al., 1983), surf clam (Suprenant and Rebhun, 1984), and cold-water fish (Langford, 1978; Williams et al., 1985; Detrich and Overton, 1986; Detrich et al., 1989; Detrich et al., 2000). These pioneering studies revealed the source-dependent variation in the intrinsic properties of the filaments (e.g., stability, critical concentration, and protofilament numbers), the optimal temperature for polymer assembly, and the responses to microtubule-destabilizing small molecules. *In vitro* polymerization of microtubules started showing the various properties of tubulin purified from different biological contexts.

Advances in the purification strategy provided high quality and enough material for analyzing tubulin polymorphism. Electrofocusing analysis of brain tubulin showed a heterogeneous mixture of a dozen polypeptides with distinct isoelectric points, suggesting the presence of subspecies of tubulin (Feit et al., 1971; George et al., 1981). Tryptic peptide mapping revealed the significant diversity of the primary sequences among tubulin subspecies (George et al., 1981). Peptide sequencing by Edman degradation further supported the heterogeneity in the primary sequences of brain tubulin, which contained at least four types of α- and two types of β-tubulin polypeptides with aspartic and glutamic residues enriched at the carboxy-terminus (i.e., the C-terminal tail) (Krauhs et al., 1981; Ponstingl et al., 1981). These studies proposed that the acidic C-terminal tails could interact with positively charged domains of microtubule-associated proteins (Krauhs et al., 1981; Ponstingl et al., 1981). Meanwhile, the cloning of mRNAs and genomic DNA analysis disclosed multiple α- and β-tubulin genes (i.e., isotypes) at different loci in the genome (Cleveland et al., 1978; Cleveland et al., 1980; Sanchez et al., 1980; Cleveland et al., 1981a). Together, the polymorphism in the tubulin primary sequences likely regulates the properties of microtubule-based cellular architectures.

### Immunofractionation of Tubulin Heterodimers With Specific β-Tubulin Isotypes

According to the molecular genetic analyses of the tubulin genes, the most divergent region between tubulin isotypes is the ∼15-residue polypeptide chain at the C-terminal tail (Sullivan and Cleveland, 1986; Villasante et al., 1986; Wang et al., 1986; Pratt et al., 1987). In particular, the amino acid sequence of the C-terminal tail is not only evolutionarily conserved across different vertebrate species but also characteristic to each β-tubulin isotype (Sullivan and Cleveland, 1986; Wang et al., 1986). While genetics and cell biology studies suggested the distinct biological functions of tubulin isotypes, the regulatory roles of tubulin isotype compositions on intrinsic microtubule properties (e.g., polymerization dynamics) was unknown due to the significant challenge of generating isotypically pure tubulin for *in vitro* biochemical and biophysical assays (Cleveland, 1987).

As the C-terminal tail has the characteristic amino acid sequence of each tubulin isotype, synthetic peptides corresponding to the tail domain can be the antigen for acquiring isotype-specific antibodies (Lopata and Cleveland, 1987). By using peptide-derived polyclonal antibodies against each of the six vertebrate β-tubulin isotypes, immunofluorescence mapped the spatial distribution of β-tubulin isotypes in cultured cells (Lopata and Cleveland, 1987). The success of this antibody-mediated approach further motivated the isolation of monoclonal antibodies for *in vitro* protein biochemistry studies of tubulin isotypes, establishing that bovine brain β-tubulin is a mixture of four subspecies: type I (β_I_, 3%), type II (β_II_, 58%), type III (β_III_, 25%) and type IV (β_IV_, 13%) (Banerjee et al., 1988). The immunodepletion of β_III_-tubulin conferred the fractionated brain tubulin an increased rate and a greater extent of microtubule assembly, suggesting the regulatory roles of tubulin isotype compositions in the microtubule polymerization properties (Banerjee et al., 1990). By employing monoclonal antibodies against β_II_-, β_III_- and β_IV_-tubulin, carefully designed immunoaffinity chromatography of bovine brain tubulin allowed the purification of tubulin heterodimers with isotypically pure β-tubulin, αβ_II_-, αβ_III_- and αβ_IV_-tubulin (Banerjee et al., 1992).

The immunofractionation of bovine brain tubulin led the way for tubulin isotypes’ functional studies. Thermodynamic characterization revealed the effects of β-tubulin isotypes on the binding affinity of antimitotic alkaloid colchicine for tubulin heterodimers (Banerjee and Luduena, 1992). Compared to the affinity for αβ_III_-tubulin, colchicine bound to αβ_II_- and αβ_IV_-tubulin about 2-fold and 30-fold tighter, respectively, (Banerjee and Luduena, 1992). In addition, the characterization of microtubule assembly showed that β-tubulin isotype compositions determined the critical concentration for polymer nucleation and the elongation behavior of the filaments (Banerjee et al., 1992; Lu and Luduena, 1994). By using differential interference contrast (DIC) video microscopy to observe single dynamic microtubules, the detailed quantification established that, in comparison to αβ_II_- and αβ_IV_-tubulin, αβ_III_-tubulin assembled into filaments with higher dynamicity (Panda et al., 1994). The compositions of β-tubulin isotypes can modulate the dynamic instability parameters (e.g., growth rate, shortening rate, and catastrophe frequency) of microtubules (Panda et al., 1994).

*In vitro* reconstitution using immunofractionated brain tubulin allowed the generation of microtubules with a defined tubulin isotype composition and opened a new avenue toward dissecting the multi-tubulin hypothesis. However, three limitations prevented the general adoption of this immunoaffinity approach for studying tubulin isotypes. First, the low variance in the C-terminal tails limits the availability of antibodies targeting specific α-tubulin isotypes. This restriction makes it challenging to understand how the crosstalk between isotypes of α- and β-tubulin determines the microtubule properties. Second, the purification of brain tubulin usually requires microtubule polymerization cycles, which are selective for tubulin isotypes that favor this process. Third, tubulin is not abundant in most non-neuronal cells. It has been challenging to achieve the critical concentration for microtubule polymerization in tubulin purification procedures. The low tubulin recovery efficiency of polymerization cycles further hinders the generation of enough tubulin from other cell or tissue types for immunofractionation. Tubulin purification strategies with higher efficiency and flexibility would be essential for dissecting the underlying molecular mechanisms by which tubulin isotypes regulate microtubule functions and structures.

### Tubulin-Affinity Chromatography For Efficient Isolation of α/β-Tubulin Heterodimers

One approach exploits tubulin-binding ligands that can be immobilized on the stationary phase as an affinity chromatography column to isolate α/β-tubulin heterodimers from complex cell extracts. In cells, conserved XMAP215/Dis1 family proteins are processive microtubule polymerases that employ the tumor overexpressed gene (TOG) domains to specifically recruit α/β-tubulin from the cytoplasm onto the growing filament ends (Al-Bassam et al., 2006; Al-Bassam et al., 2007; Brouhard et al., 2008; Widlund et al., 2011). Due to the selective and reversible binding to tubulin, the immobilized TOG domains serve as an optimal purification matrix (i.e., TOG-column) to sequester native tubulin from cell lines or tissues of various species (Widlund et al., 2012). This one-step affinity chromatography strategy allows the rapid and efficient isolation of α/β-tubulin heterodimers from extracts of cells with low tubulin expression levels, for example, *S. cerevisiae* (about 0.05% of the total protein) (Kilmartin, 1981; Widlund et al., 2012).

The success of tubulin purification using a TOG-column overcomes the following two significant drawbacks of *in vitro* biochemical reconstitution assays using mammalian brain tubulin. First, while tubulin is conserved in eukaryotes (*S. cerevisiae* and human α-tubulin protein primary sequences are about 75% identical), microtubule-associated proteins behave differently in assays with mammalian brain tubulin or with tubulin purified from homologous species (Alonso et al., 2007; Kollman et al., 2015). The TOG-column-mediated affinity chromatography strategy is revolutionary as the purified native tubulin from corresponding biological contexts is handy for homologous *in vitro* reconstitution assays. In particular, current studies have reconstituted flagellar sliding using axonemal tubulin and dynein (Alper J. et al., 2013; Alper J. D. et al., 2013; Alper et al., 2014), dissected the length regulation mechanisms of *S. cerevisiae, D. melanogaster*, or *A. thaliana* microtubules (Fujita et al., 2013; Podolski et al., 2014; Hibbel et al., 2015; Hotta et al., 2016; Moriwaki and Goshima, 2016; Otani et al., 2018; Edzuka and Goshima, 2019), elucidated the structural insight into the binding of motors and microtubule-associated proteins to *S. pombe* or human filaments (Atherton et al., 2019; von Loeffelholz et al., 2019; von Loeffelholz et al., 2017), revealed the molecular basis of the unique polymerization dynamics of *C. elegans* microtubules (Chaaban et al., 2018), identified parasite-specific small molecules targeting microtubule polymerization (Hirst et al., 2022), as well as established the roles of microtubule dynamics in the control of spindle morphology of Xenopus species (Hirst et al., 2020; Biswas et al., 2021).

Second, tubulin purified from mammalian brain tissues has various post-translational modifications (Janke and Bulinski, 2011). It has been difficult to use brain tubulin for studying the molecular mechanisms by which individual tubulin modifications regulate microtubule properties and functions. TOG-affinity chromatography allows the purification of tubulin from sources that have a clean profile of tubulin post-translational modifications, such as a human embryonic kidney cell line (tsA201) (Vemu et al., 2014; Vemu et al., 2017). The mass spectrometric analysis showed that the purified tsA201 tubulin contained α1B-, βI-, βII- and βIVB-tubulin with no detectable post-translational modification (i.e., naïve tubulin) (Vemu et al., 2014). This ‘naïve’ tsA201 tubulin can then be selectively modified by recombinant tubulin-modifying enzymes (Vemu et al., 2014). This enzymatic approach to generating filaments with a defined composition of post-translational modifications has led to mechanistic insights into the roles of tubulin’s chemical adducts in the regulation of microtubule structures and functions (Garnham et al., 2015; Valenstein and Roll-Mecak, 2016; Garnham et al., 2017; Mahalingan et al., 2020; Zheng et al., 2022).

While the TOG affinity-based approach can effectively isolate soluble tubulin from any cell lysate with no preference to specific tubulin isotypes (Widlund et al., 2012), limitations remained on characterizing how the variance in tubulin primary sequences (i.e., isotypes or mutations) could impact microtubule properties (e.g., structure, polymerization dynamics, as well as interactions with motors and microtubule-associated proteins). A recombinant protein strategy of expressing and purifying tubulin variants is essential. However, the generation of recombinant tubulin has been somewhat challenging.

### Significant Barriers to Achieving Recombinant Tubulin Stem From Its Complex Biosynthesis

*In vitro* transcription and translation system using a rabbit reticulocyte lysate system allows the purification of a small quantity of recombinant tubulin (Cleveland et al., 1978). However, achieving a yield at the milligram scale of recombinant tubulin is not trivial, likely due to the following two cellular mechanisms regulating tubulin biosynthesis and homeostasis.

First, the formation of native α/β-tubulin heterodimers requires the newly synthesized tubulin polypeptides to go through the tubulin-folding pathway that employs a complex chaperone system including prefoldin, cytosolic chaperonin, and tubulin-specific folding cofactors (Gao et al., 1992; Yaffe et al., 1992; Tian et al., 1996; Lewis et al., 1997; Tian et al., 1997; Vainberg et al., 1998; Bhamidipati et al., 2000). As common prokaryotic organisms for producing recombinant proteins (e.g., *E. coli*) lack these chaperone components, the overexpressed tubulin polypeptides of interest form non-functional aggregates in these organisms. Second, the tubulin biosynthesis is under a tight regulation that employs a negative feedback loop to self-regulate the stability of tubulin mRNAs in response to the concentration of soluble α/β-tubulin heterodimers (Ben-Ze’ev et al., 1979; Cleveland, 1989; Cleveland et al., 1981b; Gasic et al., 2019). This negative correlation, also known as tubulin autoregulation, involves a ribosome-associating factor, TTC5, that binds to the N-terminus of nascent tubulin polypeptides and stimulates co-translational degradation of tubulin mRNA following increased soluble tubulin concentration (Yen et al., 1988; Theodorakis and Cleveland, 1992; Lin et al., 2020).

Together, due to the complex eukaryotic machinery that controls the folding and the concentration of soluble tubulin in the cytoplasm, the yield of recombinant tubulin is irrelevant to the ectopic overexpression level of the tubulin genes of interest. It has been challenging to generate recombinant tubulin in the native state for dissecting how variation in the primary protein sequences affects the structures and functions of microtubules.

### Exploiting Yeast Cells to Express and Purify Recombinant Tubulin

The genomes of budding yeast *S. cerevisiae* and fission yeast *S. pombe* encode two α- and one β-tubulin isotypes, which the yeast genetic tools can engineer to construct strains harboring modified tubulin genes. With the established methodology to purify milligram quantities of wild-type yeast tubulin proteins (Barnes et al., 1992; Davis et al., 1993), the relatively simple tubulin isotype composition has made these unicellular fungi a powerful platform to access mutant tubulin proteins. In particular, the stable haploid and diploid states of yeast cells provide an opportunity to characterize tubulin harboring lethal mutations (Davis et al., 1994). Furthermore, the genetically modified strains with only one α-tubulin isotype have been the source for generating isotypically pure yeast α/β-tubulin heterodimers, which allows the assembly of microtubules with a defined tubulin isotype composition (Bode et al., 2003; Braun et al., 2009; des Georges et al., 2008; Uchimura et al., 2010; Uchimura et al., 2006).

To isolate the mutant tubulin from yeast cells for *in vitro* assays, researchers engineered the yeast β-tubulin C-terminus to include a hexahistidine tag for affinity purification or to alter the quantities of negatively charged glutamic acid residues for ion-exchange chromatography (Davis et al., 1994; Gupta et al., 2002). These yeast strains expressed α- and β-tubulin proteins from the endogenous gene loci or extra copies of the α- and β-tubulin genes controlled by a galactose-induced overexpression promoter. The chromatography-based strategy and affinity purification allowed the isolation of mutant tubulin without cycles of polymerization and depolymerization (Davis et al., 1994; Gupta et al., 2002). The mutagenesis analyses of yeast tubulin have provided mechanistic insights into the roles of GTP hydrolysis activity in microtubule dynamics (Davis et al., 1994; Dougherty et al., 2001), investigated the structure-activity relationship of tubulin-targeting small molecules (Gupta et al., 2002; Gupta et al., 2003), and examined the microtubule-binding site regulating the kinesin motor activity (Uchimura et al., 2006; Uchimura et al., 2010). However, the excess amounts of β-tubulin (alone or together with α-tubulin) causes cell cycle arrest, chromosome losses, and depolymerization of cellular microtubules (Burke et al., 1989). The lethality limited the yield of recombinant yeast tubulin and restricted access to dominant loss-of-function mutant tubulin for *in vitro* biochemical and biophysical characterization. The full capacity of the yeast protein expression system remained unexplored.

A robust strategy has unleashed the power of using *S. cerevisiae* to express and purify recombinant mutant tubulin (Johnson et al., 2011). The success of this chromatography-based approach depends on a transient (three to five hours) but strong protein overexpression from galactose-inducible promoters in high-copy-number plasmids. By significantly improving the final yield while bypassing the lethality due to excess α- and β-tubulin, this strategy opens a new avenue to characterizing tubulin constructs harboring dominant polymerization-blocking mutations, which have explicated the principles of protein machinery that regulates microtubule growth and stability (Ayaz et al., 2012; Ayaz et al., 2014; Geyer et al., 2018; Majumdar et al., 2018). Further mutagenesis analyses of recombinant yeast tubulin revealed the long-overdue molecular bases of microtubule dynamics, such as the allosteric effects of nucleotide states and the regulatory roles of nucleotide exchange on growing filament ends (Geyer et al., 2015; Piedra et al., 2016), demonstrated the effects of disease-related tubulin mutations on microtubule properties (Denarier et al., 2021; Park et al., 2021), and elucidated the mechanism of kinesin-8 family depolymerase activity (Arellano-Santoyo et al., 2017).

This established recombinant yeast tubulin system also has stimulated the development of new methodologies to label tubulin for revealing molecular features of microtubules. For example, site- and topology-specifically labeled yeast tubulin with probes or tags can facilitate studies of how proteins, small molecules, and post-translation modifications interact and regulate microtubules (Kleiner et al., 2013). Furthermore, strategies tagging the C-terminus of yeast β-tubulin with a microbead or a gold nanoparticle have significantly improved the resolution of light microscopy for characterizing *in vitro* reconstituted microtubules (Driver et al., 2017; Mickolajczyk et al., 2019). Direct measurements by laser tweezers determined the strain energy stored in microtubule protofilaments (Driver et al., 2017), while direct observation of tubulin subunits association and dissociation at growing filament ends provided quantitative insights into microtubule dynamics (Mickolajczyk et al., 2019).

Using this yeast-based strategy to generate recombinant human tubulin was unsuccessful (Sirajuddin et al., 2014). Instead, an alternative approach employed chimeric proteins consisting of the folded yeast tubulin core and the human tubulin unstructured C-terminal tail that contains sites for several post-translational modifications and interacts with most microtubule-associated proteins (Sirajuddin et al., 2014; Janke and Magiera, 2020). These chimeric tubulin proteins were purified by affinity chromatography using a hexahistidine-tag in the acetylation loop of the α-tubulin subunit. These tubulin chimeras have been a powerful tool to characterize the regulatory roles of tubulin isotypes and post-translational modifications in protein activities such as the processivity and velocity of microtubule motors (Sirajuddin et al., 2014) and the permeability of voltage-dependent anion channels (Rostovtseva et al., 2018).

### The Recent Breakthrough in Making Recombinant Higher Eukaryotic Tubulin

The genome of higher eukaryotes encodes a substantially expanded number of α- and β-tubulin isotypes. For example, the human genome encodes at least nine α- and ten β-tubulin isotypes that show cell-type-specific expression profiles (Ludueña and Banerjee, 2008; Findeisen et al., 2014). While genetics and cell biology studies suggested that each tubulin gene could have unique cellular functions (Janke and Magiera, 2020), it remains unclear how tubulin isotypes modulate microtubule structures and functions. Due to the complex cellular machinery that regulates tubulin biosynthesis and homeostasis, it has been challenging to purify active human tubulin in the recombinant form.

In 2013, a pioneering strategy used the baculovirus-insect cell protein expression system to generate polymerization competent recombinant human tubulin (Minoura et al., 2013). By employing a hexahistidine-tag fused to the C-terminus of α-tubulin and a FLAG-tag at the C-terminus of β-tubulin, this affinity chromatography-based workflow allowed co-expressing both tubulin and generating high purity of active human α1/β3-tubulin, mouse α1/β2-tubulin and Drosophila α1/β1-tubulin (Minoura et al., 2013; Ayukawa et al., 2021; Diao et al., 2021). Later, another approach used an internal hexahistidine-tag at α-tubulin (Sirajuddin et al., 2014) together with a protease-cleavable FLAG-tag at the C-terminus of β-tubulin also successfully produced isotypically pure human α1/β3-tubulin (Vemu et al., 2016). The success in generating active α/β-tubulin isotypes of higher eukaryotes has offered the opportunities to dissect the molecular mechanisms by which high eukaryotic tubulins regulate microtubule properties. In particular, recombinant human α1/β3-tubulin has become a popular material for characterizing the impacts of disease-related tubulin mutations on the behaviors of kinesin motors (Minoura et al., 2016), the modulation of microtubule dynamics by tubulin isotype composition (Vemu et al., 2017), the incorporation of soluble GTP-tubulin into damaged sites along the microtubule shaft (Vemu et al., 2018), and the effects of GTP hydrolysis on microtubule structures and dynamics (Roostalu et al., 2020; LaFrance et al., 2022). However, these recombinant proteins contain uncleavable charged affinity tags fused to the tubulin domains that interact with microtubule-associated proteins (e.g., the C-terminal tail) or inter-tubulin contacts within the microtubule lattice (e.g., the acetylation loop). It will be favorable to access recombinant tubulin with cleavable affinity tags.

Tubulin with a cleavable affinity tag can be a powerful tool for identifying tubulin-associated proteins (Yu et al., 2016; Yu and Galjart, 2018). A SUMO protease cleavable biotinylation tag at the N-terminus of human α1-or β3-tubulin mediated the isolation of tubulin and the associated tubulin-binding proteins from the cell lysate (Yu et al., 2016; Yu and Galjart, 2018). The expression of the tubulin constructs together with bacterial biotin ligase (BirA) in HEK293T cells led to the generation of biotinylated human tubulin. After streptavidin-coupled matrix-based enrichment, SUMO protease treatment facilitated the release of biotin-tagged tubulin and the tubulin-associated proteins for further mass spectrometry analyses. While this approach provides an opportunity to reveal the interaction proteome of human tubulin isotypes or disease-related mutant tubulins (Yu and Galjart, 2018), the relatively low yield has limited the application of this strategy to generate recombinant human tubulin for *in vitro* reconstitution of microtubules.

To obtain affinity tag-free recombinant tubulin, we reasoned that the cleavable affinity tag must be at the N- or C-terminus of tubulin for enzymatic removal of the peptide tag after affinity chromatography. While it is promising to fuse the affinity peptide ligand to the C-terminus of β-tubulin, the initial attempts to tag either end of α-tubulin significantly reduced the amount of recombinant human tubulin obtained. By employing the baculovirus-insect cell system, we expressed untagged human α1B-tubulin together with human β2-or β3-tubulin fused at the C-terminus with a tobacco etch virus (TEV) protease cleavable hexahistidine-tag (Ti et al., 2016). Our three-step tubulin purification strategy involved nickel-affinity chromatography followed by tag removal and the final TOG-column affinity chromatography. This approach yielded isotypically pure human β-tubulin dimerized with either recombinant human α1B-tubulin or endogenous insect α-tubulin, indicating that the C-terminal hexahistidine tag is sufficient to isolate specifically human β-tubulin from the complex cell lysate. The characterization of the isotypically pure recombinant human β-tubulin revealed how disease-related β-tubulin mutations, human β-tubulin isotypes, and tubulin allosteric conformational changes affect microtubule dynamics (Pamula et al., 2016; Ti et al., 2016; Ye et al., 2020).

Our systematic evaluation indicated that both the composition and position (N- or C-terminus) of the fused polypeptide are critical for the yield of functional human tubulin. To achieve optimal cleavage efficiency of the affinity tags, we incorporated a TEV-cleavable decahistidine tag with an Ala-Pro dipeptide linker to the N-terminus of human α1B-tubulin and a TEV-cleavable strep tag with a Gly-Gly-Ser-Gly-Gly pentapeptide linker to the C-terminus of human β2-and β3-tubulin (Ti et al., 2018; Ti et al., 2020). We note that the enzymatic digestion gets rid of the affinity tags but leaves residual ‘scars’ at the N-terminus of the α-tubulin (Gly-Ala-Pro) and the C-terminus of the β-tubulin (Glu-Asn-Leu-Tyr-Phe-Gln). We speculate that combining our approach with other protein engineering tools (e.g., protein ligation) will generate recombinant human tubulin with native sequence.

With these constructs, we recently developed an affinity chromatography-based purification strategy that allows the routine preparation of affinity tag-free recombinant human tubulin. As the sequential isolation of human α- and β-tubulin depends solely on the affinity tags, this approach applies to studies of tubulin variants (e.g., isotypes and mutants) that could impact the binding to TOG domains or the microtubule polymerization properties. By employing this strategy, current studies have revealed the effects of human β-tubulin isotypes on the microtubule stability and protofilament numbers (Ti et al., 2018) as well as dissected the molecular mechanisms by which methyltransferases modify human α-tubulin (Kearns et al., 2021). Together, the ability to obtain biochemically pure higher eukaryotic tubulin has paved the way to deciphering the functions of tubulin diversity and a clearer understanding of microtubule biology.

## Conclusion and Perspectives

**FIGURE 3:**
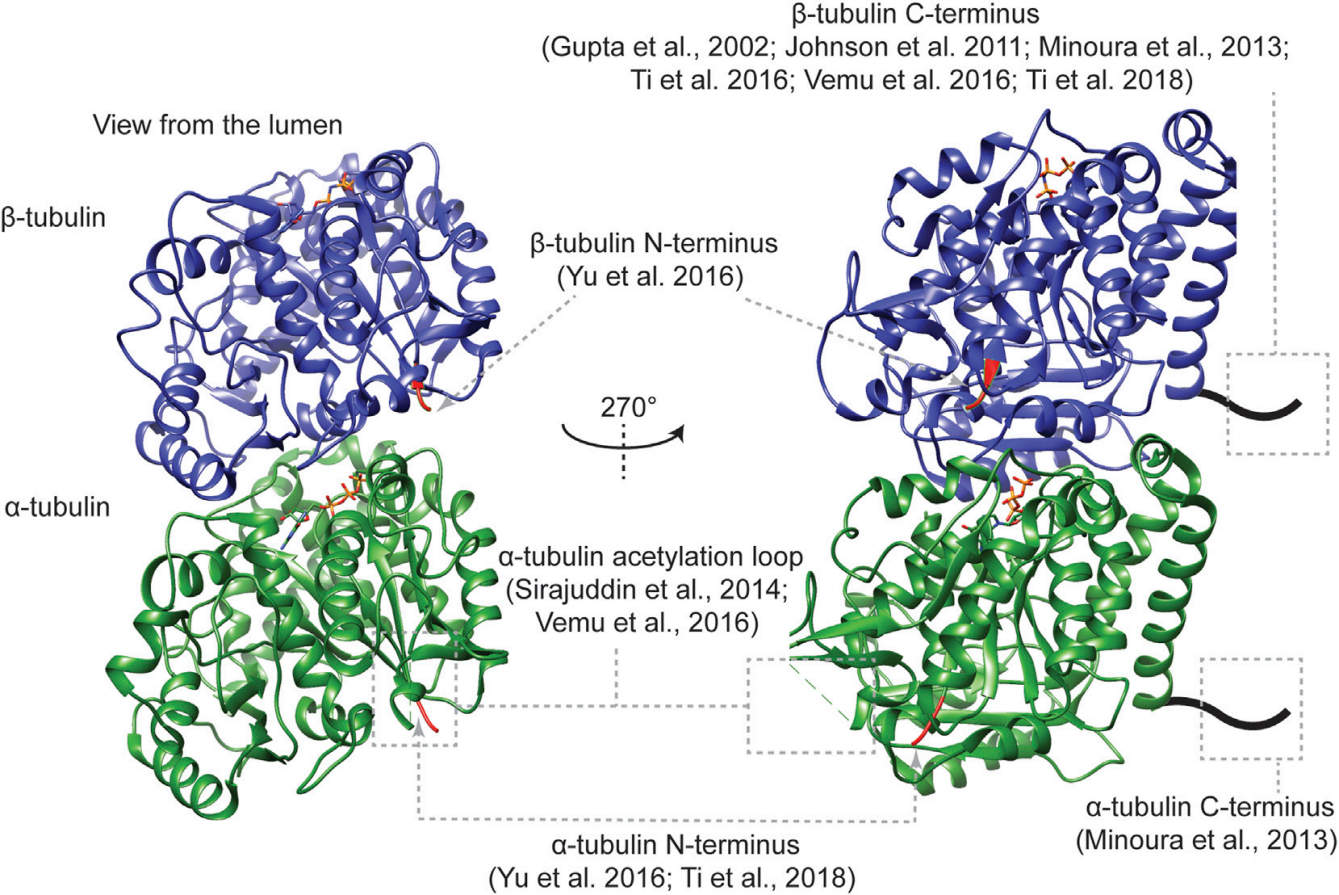
The location of affinity tags for the purification of recombinant tubulin. The ribbon diagrams show the structure of an α/β-tubulin heterodimer (PDB ID: 6e7b). The first three residues at the N-termini of each tubulin are in red, while the rest of α- and β-tubulin are in green and blue, respectively. The stick model represents the tubulin-bound nucleotides.

Tubulin protein biochemistry has been evolving since more than 50 years ago, when the colchicine-binding activity led to the isolation of the building blocks of endogenous microtubules (Borisy and Taylor, 1967; Shelanski and Taylor, 1967). With recently established affinity tag-based strategies of generating recombinant α/β-tubulin with defined primary sequences (Figure 3), it becomes feasible to correlate *in vivo* tubulin mutagenesis analyses with *in vitro* biochemical and biophysical characterization of mutant tubulin. This integrative approach is potentially applicable to the mutagenesis analysis of any tubulin isotype of interest for a mechanistic understanding of how tubulin diversity regulates cellular microtubule structures and functions.

The advances in tubulin protein biochemistry also provide opportunities to address some fundamental questions in microtubule biology by developing the needed tools such as 1) isotype-specific antibodies/nanobodies to characterize the spatial distribution of tubulin isotypes in cellular microtubules, 2) recombinant tubulin incorporated with a probe at a specific site for the identification of small molecules or protein binders targeting explicit tubulin isotypes, 3) engineered tubulin harboring defined modifications to investigate the crosstalk between tubulin isotypes and post-translation modifications (i.e., the tubulin code), and 4) small molecules targeting tubulin isotypes of interest not only for dissecting the biological functions but also for novel chemotherapeutic agents. By combining recombinant tubulin with a chemical biology toolbox for protein engineering (e.g., amber suppression and protein ligation), these technology breakthroughs will expand our ability to tackle the challenges in the field. We speculate that decades of research have set the stage to unveil the molecular basis of how cells establish and use the heterogeneous microtubule composition to facilitate the functional outputs.
